# Good care for older people at the end of life: Shared responsibilities, flexible boundaries

**DOI:** 10.1016/j.fhj.2026.100515

**Published:** 2026-03-27

**Authors:** Felicity Dewhurst, Polly M. Edmonds, Rowan H. Harwood, Daniel S. Furmedge

**Affiliations:** a Population Health Sciences Institute, Newcastle University, Newcastle upon Tyne, UK; b St Oswald’s Hospice, Gosforth, Newcastle upon Tyne, UK; c Department of Palliative Care, King’s College Hospital, London, UK; d School of Health Sciences, University of Nottingham, Nottingham, UK; e Centre for Ageing Resilience in a Changing Environment (CARICE), King’s College London, London, UK

**Keywords:** Older people, Palliative care, End of life care, Frailty, Multimorbidity

## Abstract

The majority of deaths occur among older adults, many living with frailty, multimorbidity, disability and/or cognitive impairment. When care is organised around single diseases and episodic crises, people can experience fragmented, reactive care and burdensome interventions. We argue that good end-of-life care is a shared responsibility across settings, requiring flexible boundaries between geriatric medicine, palliative care, primary care, social care and the voluntary sector. Clinical vignettes illustrate challenges including prognostic uncertainty, treatment burden, transitions between services, and achieving preferred place of care and death. We propose neighbourhood-based, person-centred care that anticipates deterioration: shared decision-making and parallel planning; minimising treatment burden (including deprescribing); coordinated anticipatory care plans with accessible records; and timely care in the last months focused on comfort, dignity and family support. Delivery depends on appropriate funding and workforce capacity.

## Older people are at risk of dying

Most people die in older age.^1^ All older people are, to some extent, approaching the ends of their lives. Older peoples’ health is associated with medical complexity, multimorbidity, frailty, disability, dependency and cognitive impairment.^2^, ^3^, ^4^ The possible approach of the end of someone’s life should inform all aspects of older peoples’ healthcare. Population ageing and fragile social structures are exponentially increasing need.

Care providers strive to get care at the end of life right, working within services that are sometimes reactive, fragmented, single disease-centred and stretched.^5^, ^6^, ^7^, ^8^ Historically, families and communities were integral to the dying process, but knowledge, skills and traditions in this context have been lost.^9^ Health and social care professionals are increasingly required to support people to navigate ‘normal dying’.

The characteristic feature of frailty is propensity to crises, sudden deteriorations, including infections, adverse drug effects, falls and delirium, which are common.^10^ Frail, older people can have multiple symptoms: pain, fatigue, anorexia and breathlessness are common. However, generally functional issues are more troublesome; these include immobility, falls, incontinence, poor oral intake, agitation or behavioural problems. Disability and dependency require support, from families, professional care or care homes; cost and carer strain are concerns.

When asked, bereaved carers identified key issues affecting their experience of care, including difficulties in accessing services, lack of personalised care, poor coordination and inadequate communication between providers.^11^

How can the insights and expertise of geriatric medicine and palliative care help us to address older peoples’ needs at the end of life?

## Challenges with current care provision

**Fig. 1:**
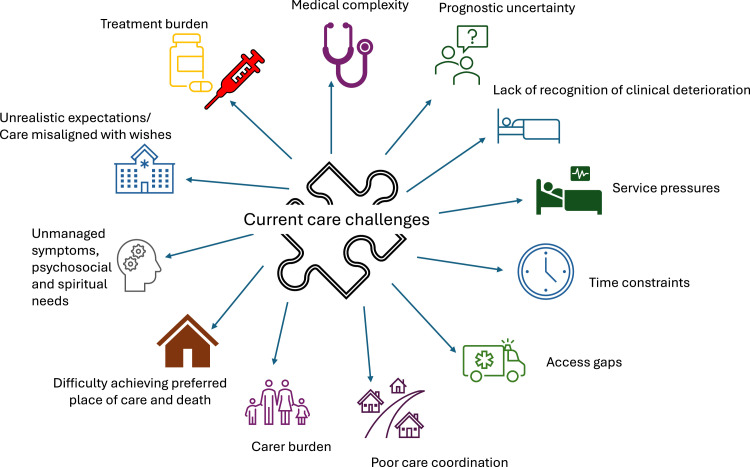
Challenges with current care provision.

Several factors create challenges to care provision (Fig. 1).

### Clinical deterioration and unmanaged expectations

In cancer, there may be a clear point when the focus of treatment shifts to palliation. With older people, this may sometimes be the case (eg very advanced dementia), but for many, there is no specific point that indicates someone is dying. Standard geriatric medical care is problem-orientated, most is ‘supportive’ and much is ‘palliative’.

Deterioration at older age can follow a dramatically fluctuating course, with periods of stability interspersed with decline associated with acute illness or injury, which can recover to a greater or lesser extent. Older people often look like they are dying when ill, especially if they have hypoactive delirium, and it can be difficult to predict the outcome for a given individual during a particular episode.

The last years of life for older people can be characterised by uncertainty over prognosis and appropriate management with the potential for decision-paralysis and conflict between teams, older adults and those close to them. While some uncertainties may be resolved with flexible, proactive communication and care provision, other uncertainties, such as future needs and prognosis, often remain, and can be challenging for care providers to manage.^12^

### Treatment burden

Case 1 **Treatment burden, exacerbated by socioeconomic deprivation: Keith.**

**Keith, 67,** r**etired** l**abourer, Byker, Newcastle**

**Diagnoses: Severe COPD, heart failure, diabetes and previous stroke with mobility impairment**

Keith lived alone in one of the most socially deprived areas in England. He has had several admissions with type 2 respiratory failure, which led to initiation of home non-invasive ventilation and long-term oxygen therapy with limited consideration of his preferences and social circumstances. At home, Keith became dependent on the ventilator. Fatigue and breathlessness meant that he rarely took it off, developing malnutrition and facial pressure sores. He was too scared to use his oxygen as he could smell his neighbour smoking. His prepayment electricity meter frequently ran out, causing the ventilator to alarm, leading to constant anxiety and frequent calls to emergency services.

Despite subsequent support from district nursing and the community response team, Keith had lost his confidence and ability to be at home, feeling trapped and exhausted. He was admitted to a hospice for end-of-life care, where symptom control allowed him to enjoy a few sips of beer while watching the football before dying a few days later.

Alt-text: Unlabelled box dummy alt text

All medical care carries a cost.^13^ As specialist treatments develop and generalism becomes increasingly fragile, the concept of what may be reversible is increasingly blurred. Defensive practice is common. Sometimes sight of the bigger picture is lost.

Treatments may cause adverse effects and disruption, require effort and time, and have a financial cost.^14^^,^^15^ People living with frailty experience greater burdens, which can overwhelm people and families, particularly in socioeconomically deprived areas, where transport, housing, heating and food insecurity are also daily concerns.

Frail older people are exquisitely prone to adverse drug effects, especially of opioids, antipsychotic and anticholinergic drugs. Clinicians need to be bolder in deprescribing, asking what the benefits and risks of each medication really are.^16^ It is far easier to prescribe than to stop a medicine.

Recognising treatment as a potential harm and explicitly weighing it against potential benefit, in the context of a person’s individual preferences, is central to decision-making, but also requires confidence and skill on the part of the clinician.

### Service pressures

The scale of need is rising fast, putting significant pressure on primary and secondary care, adult social care and the voluntary sector,^5^, ^6^, ^7^ with gaps in proactive identification, timely response, variable access to multidisciplinary community provision, and persistent fragility in ‘out of hours’ response and skill mix. All contribute to potentially avoidable escalation into hospital at times of deterioration.^5^, ^6^, ^7^^,^^17^ Stretched services at capacity may ‘reject’ referrals, for example a proactive palliative care referral is rejected due to insufficient capacity and no ‘active palliative care needs’, which may lead to missed opportunity before crisis.

Continuity and integration of care are valued by patients, but they are often missing in fragmented services designed to be reactive, episodic or even competitive, with restrictive eligibility criteria and inadequate inter-service collaboration and communication. Integrated IT systems play a crucial role in reducing fragmentation by enabling timely information sharing, interoperable electronic records and data‑driven care coordination^18^^,^^19^ (Case 2).

Case 2 **Service pressures: Fred.**

**Fred, 85,** r**etired dockworker, Portsmouth**

**Diagnoses: End**-**stage heart failure, COPD and CKD**

Fred had six admissions in 6 months with fluid overload, cardiorenal syndrome and frailty, under cardiology, general medicine and geriatric medicine across two different hospitals. He found the hospital environment challenging and wanted to be at home. Each time fluid was offloaded and Fred was discharged with increased care.

On his final admission, he was referred to hospital at home for intravenous diuresis and therapy input. He continued to deteriorate, was recognised as dying by the hospital at home geriatrician and his care was transferred to the palliative care virtual ward. His furosemide was switched to a subcutaneous infusion along with alfentanil and midazolam in his last days of life. He died at home, away from the noise and bustle of the hospital which he hated.

Alt-text: Unlabelled box dummy alt text

### Place of death is challenging

Dying at home is often held as an ideal of good end-of-life care, but the reality can be very challenging. Dying at home presumes that the person lives in a suitable physical environment. Informal carers often need to cover gaps in formal care provision and can feel isolated and responsible. Older family members may themselves be frail or disabled. Issues with timely access to equipment, support, medication and advice cause significant carer burden and may lead to hospital admission.

For people who wish to die at home, improvements must be made to allow this to happen. For those who don’t, or can’t, hospitals, care homes and hospices should have the capacity and resource to allow for high-quality care of the dying.

## What does good look like?

The fundamental aspects of good care towards the end of life already exist within current teams and services: excellent clinical skills, personalised care provision, timely response and good communication with shared decision-making.

**Fig. 2:**
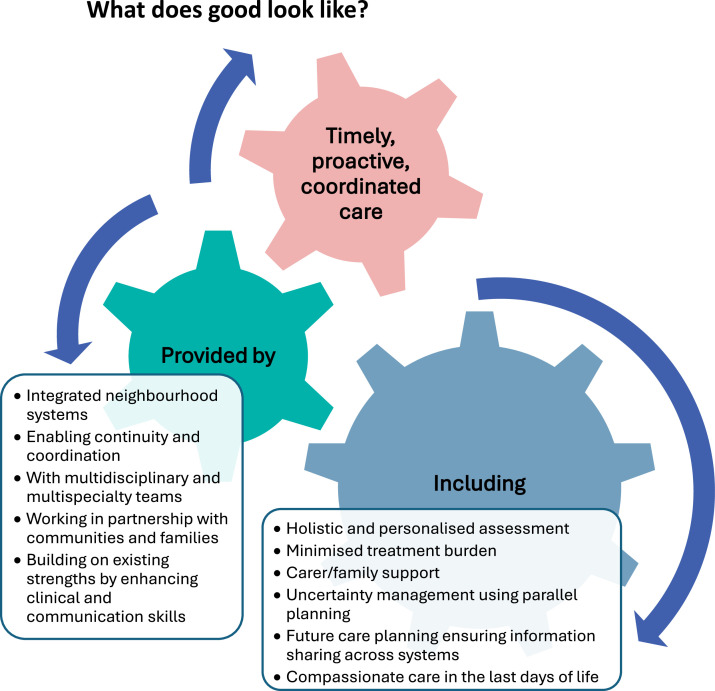
Proactive, coordinated care to navigate dying.

Managing chronic and progressive disease requires a holistic approach; in older people, this is known as ‘Comprehensive Geriatric Assessment’^20^:

• Medical treatment, optimisation or disease modification
• Rehabilitation
• Mental healthcare, and concern for psychological and emotional wellbeing
• Social and environmental interventions, including supporting carers.

Challenges in prognostication may be supported by approaches such as ‘parallel planning’.^21^^,^^22^ This requires making different plans for both more and less aggressive medical care, but requires services to scaffold and be available to respond appropriately.

A consequence of uncertainty is the need to support individuals to ‘live well’ at the same time as being realistic about the approaching end of life. This parallel planning requires a focus on retaining and maximising abilities, concentrating on ‘what matters’ for the individual, optimising the environment, ensuring opportunity and social inclusion, and supporting family and other carers.

A palliative approach can improve patient and family outcomes and reduce costs,^23^ with multidisciplinary, multicomponent and multi-setting models being most effective.^24^ There is most benefit when needs-based care is received at least 3 months before death.^24^ While many older, frail people do not require a palliative care specialist, they do benefit from a holistic assessment of needs, care planning and coordination, including:

• Proactive management of symptoms, needs and distress
• Maintenance of function, enablement and wellbeing
• Reducing the burden of intervention
• Making plans for the future
• Timely referral to specialist services for those with more complex requirements.

**Fig. 3:**
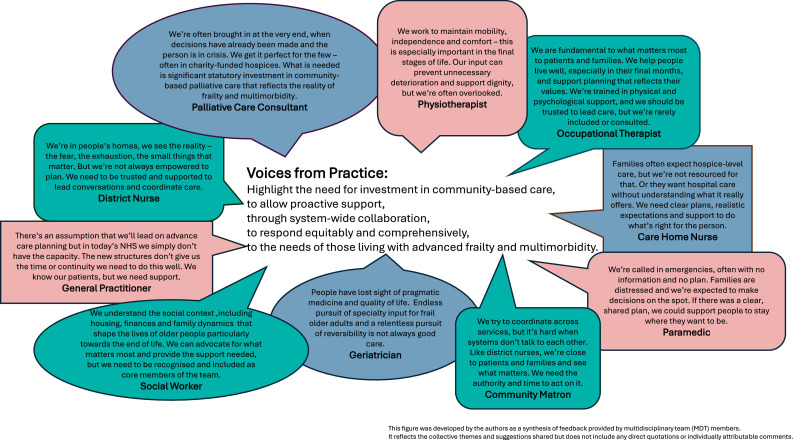
Voices from practice.

This holistic approach requires input from a multidisciplinary team, delivering continuity and coordination where possible. Across all care settings, a range of professionals, including general practitioners, geriatricians, palliative care, old age psychiatry, allied health professionals and specialist nursing, can provide support: the key is getting the right expertise for a given problem and skilled senior decision makers available at a time of crisis.^25^ Fig. 3 shares some voices from practice (quotes have been used for illustration purposes and are not attributable to individuals).

Neighbourhood health may offer a unifying framework for delivering high-quality care.^28^ By integrating primary care, social care, care homes, voluntary services, and specialist input within local systems, neighbourhood health models aim to overcome fragmentation and promote continuity.^17^^,^^26^, ^27^, ^28^

This approach requires capacity and capability, which will require significant shifts in funding to the community – in theory a key priority for government, with action yet to be seen. Embedding palliative care and geriatric medicine within neighbourhood health systems is a critical step towards person-centred, sustainable care for older adults with frailty.

### Minimising treatment burden

‘Minimally disruptive medicine’ (or ‘realistic medicine’) is an approach that focuses on individual preferences, takes account of multimorbidity and frailty, and seeks to reduce the workload for patients and caregivers.^13^^,^^29^ Minimising treatment burden by stopping, not starting or not escalating interventions, including diagnostic investigations, requires time, explanation and negotiation. Some burdens may be minimised by providing care in community settings, including care homes, virtual wards and hospital at home.^17^^,^^25^^,^^26^

### Future care planning

Personalised care requires honest, compassionate, culturally sensitive communication and an ability to ‘hold’ uncertainty. The use of Comprehensive Geriatric Assessment^20^ or tools such as the Integrated Palliative Outcome Scale^30^ can optimise person-centred management and improve patient outcomes. Tools which identify problems also require plans and resources to meet them, and do not replace therapeutic relationships based on high-quality skilled communication.

Advance care planning (ACP) is a core element of proactive care. It provides an opportunity for people to say what matters most to them, explore options for care towards the end of life, state what they would and would not want, and identify who should speak for them if they cannot speak for themselves. An ACP conversation should be offered but not imposed on people, framed as a chance to get things right if capacity is lost and to ease the stress of future crises, rather than as a limitation of treatment. It works best as a series of conversations, supported by an evolving record that can be updated as health and circumstances change (Case 3).

Case 3 **Advance care planning: Bertha.**

**Bertha, 78,** r**etired factory worker,** s**outh London**

**Diagnosis: Corticobasal degeneration**

Bertha had a long admission with severe pneumonia with a permanent decline in function, cognition and swallow with constant screaming. The medical team tried to avoid a gastrostomy, but this was inserted due to her family’s strong preference. DNACPR discussions were challenging. Her family struggled to accept her poor prognosis with cultural views around sanctity of life.

Bertha moved to a nursing home. The initial months were difficult, but staff gained trust by addressing some longstanding issues that the family had felt had been neglected previously. Her screaming mostly resolved.

The care home MDT felt that Bertha would be best managed within the care home setting, but initial discussions to enable this were not fruitful. After 6 months of trust building, a meeting between the care home MDT, including geriatrician, GP and nursing team, allowed an advance care plan to be agreed – having built confidence, Bertha’s family agreed that she would not want to go back to hospital. She was treated twice for aspiration pneumonia in the care home. On the third episode she died there – 14 months after arrival.

Alt-text: Unlabelled box dummy alt text

Examples of advance care planning include advisory documents, such as DNACPR (do not attempt cardiopulmonary resuscitation), advance statements, emergency healthcare and treatment escalation plans, and legally binding documents such as an advance decision to refuse treatment. Appointment of a legal proxy decision maker can also ensure that preferences are respected. When plans are absent or inaccessible, relatives may be left feeling that they carry a burden of decision making without knowing what the person wanted, and clinicians who have never met the person may default to burdensome interventions.^31^ Preferences and wishes should be accessible to all healthcare providers. Some areas have well-developed electronic care coordination systems,^32^ such as London’s Universal Care Plan^33^ and the ReSPECT process;^34^ these should be evaluated and replicated as appropriate.

Staff may lack confidence initiating future-focused conversations and may need targeted training and opportunities for mutual support. Many services focused on management of chronic diseases that commonly lead to death, such as heart failure or COPD teams, routinely appear to omit advance care planning; this should be challenged and barriers addressed. Strengthening skills and confidence, alongside continuity of care that builds trust, enables better shared decision-making.

### Care in the last days of life

When death is anticipated in hours or days, attention should be directed towards comfort, dignity, avoidance of burden and support for family. Care needs to be proactive and timely to address needs. In general, as oral intake reduces, medications that are not essential for symptom control can be discontinued. Explanations to patients (if able) and families, to help them understand the normal dying process, can help to reduce distress.

The use of clinically assisted nutrition and hydration should be reviewed on a case-by-case basis; for most people, the burden of continuing will outweigh benefits.

Many patients will benefit from having medication for common symptoms in the dying phase prescribed for use as needed (frail patients often require dose reductions). Some will require a continuous subcutaneous infusion for symptom management.

### Families and communities as partners in care

Families, or those close to a person, are vital partners in care (Case 4). Community understanding of dying (‘death literacy’) underpins realistic and compassionate conversations. Innovations such as death doulas, community networks and death cafés contribute to building capacity that helps normalise talking about death and dying.^9^^,^^35^ There is evidence that community engagement initiatives improve health and social care outcomes at the end of life.^36^^,^^37^

Case 4 **Families and communities as partners in care: Amina.**

**Amina, 82,** f**amily** m**atriarch, Bolton**

**Diagnoses: Severe frailty, heart failure, type 2 diabetes, osteoarthritis**

After several years of support from family, an unplanned admission with sepsis with functional decline led to a community matron visit to explore what was important to Amina and her family. Amina did not want to discuss death, stating, ‘only Allah will decide’. The matron acknowledged this and asked what a good day looked like and what would worry them the most. Being at home, being able to pray, hearing Qur’an recitation and having female carers mattered more than being kept alive. A Muslim chaplain helped explain that planning ahead was not about controlling the time of death, but ensuring that religious beliefs and practices could be respected when death came. They agreed that she would not be for critical care or CPR and that the focus would be on treatment and symptom control at home. She asked that her son could speak for her and her wishes if she could not. A treatment escalation plan was developed and placed on the locally shared record, which included information about religious preferences, such as turning the bed towards Mecca (the Qibla) if she was thought to be dying.

8 months later she developed pneumonia. Using the plan, she was cared for at home with appropriate nursing and chaplaincy. She died peacefully at home with her family around her and her wishes and religious faith fulfilled.

Alt-text: Unlabelled box dummy alt text

## Conclusion

High-quality end-of-life care is core business across health and social care, requiring a coordinated, system-wide approach that makes good care the default, not the exception.

Excellent care requires awareness that individual patients may be dying and improvements in societal death literacy, normalising conversations regarding death, dying, preferences and wishes. High-quality communication is needed to avoid unwanted and futile medical care, while enabling wanted medical care that addresses problems at tolerable cost.

Dying at home is an ideal for many but is not always possible. Home assessment and treatment of crises, including out-of-hours, can help avoid unnecessary or unwanted admission to hospital; this requires strengthening community capability to respond, including care home outreach, hospital at home, rehabilitation and carer support. Some admissions are unavoidable, and hospital clinicians also need to be skilled in assessment, decision making and planning, including navigating the balance between potential reversibility and futility. Hospitals and care homes need to be good places to die, if dying at home is not an option. Hospice beds need to be invested in and expanded.

Specialist palliative and geriatric medical care should be embedded across settings as an enabling function – supporting clinical teams, complex symptom management, communication and decision-making – rather than a late-stage referral, with equitable access for non-cancer populations. Services should be locality based and work together rather than in silos. Furthermore, services need to measure what matters and learn quickly, using patient-centred outcomes (symptom burden, function and carer strain, preferred place of care/death), and data on service use optimisation (avoided clinic attendances, admissions and emergency care) to drive continuous improvement and investment decisions.

## CRediT authorship contribution statement

**Felicity Dewhurst:** Writing – review & editing, Writing – original draft, Conceptualization. **Polly M. Edmonds:** Writing – review & editing, Writing – original draft, Conceptualization. **Rowan H. Harwood:** Writing – review & editing, Writing – original draft, Conceptualization. **Daniel S. Furmedge:** Writing – review & editing, Writing – original draft, Conceptualization.

## Declaration of competing interest

The authors declare that they have no known competing financial interests or personal relationships that could have appeared to influence the work reported in this paper.
